# Wire-like Pt on mesoporous Ti_0.7_W_0.3_O_2_ Nanomaterial with Compelling Electro-Activity for Effective Alcohol Electro-Oxidation

**DOI:** 10.1038/s41598-019-51235-4

**Published:** 2019-10-15

**Authors:** Hau Quoc Pham, Tai Thien Huynh, Anh Tram Ngoc Mai, Thang Manh Ngo, Long Giang Bach, Van Thi Thanh Ho

**Affiliations:** 1 Ho Chi Minh City University of Technology, VNU-HCM, Ho Chi Minh City, Vietnam; 2 Hochiminh City University of Natural Resources and Environment (HCMUMRE), Ho Chi Minh City, Vietnam; 3 NTT Hi-Tech Institute, Nguyen Tat Thanh University, Ho Chi Minh City, Vietnam

**Keywords:** Fuel cells, Fuel cells

## Abstract

Finding out robust active and sustainable catalyst towards alcohol electro-oxidation reaction is major challenges for large-scale commercialization of direct alcohol fuel cells. Herein, a robust Pt nanowires (NWs)/Ti_0.7_W_0.3_O_2_ electrocatalyst, as the coherency of using non-carbon catalyst support and controlling the morphology and structure of the Pt nanocatalyst, was fabricated via an effortless chemical reduction reaction approach at room temperature without using surfactant/stabilizers or template to assemble an anodic electrocatalyst towards methanol electro-oxidation reaction (MOR) and ethanol electro-oxidation reaction (EOR). These observational results demonstrated that the Pt NWs/Ti_0.7_W_0.3_O_2_ electrocatalyst is an intriguing anodic electrocatalyst, which can alter the state-of-the-art Pt NPs/C catalyst. Compared with the conventional Pt NPs/C electrocatalyst, the Pt NWs/Ti_0.7_W_0.3_O_2_ electrocatalyst exhibited the lower onset potential (~0.1 V for MOR and ~0.2 for EOR), higher mass activity (~355.29 mA/mg_Pt_ for MOR and ~325.01 mA/mg_Pt_ for EOR) and much greater durability. The outperformance of the Pt NWs/Ti_0.7_W_0.3_O_2_ electrocatalyst is ascribable to the merits of the anisotropic one-dimensional Pt nanostructure and the mesoporous Ti_0.7_W_0.3_O_2_ support along with the synergistic effects between the Ti_0.7_W_0.3_O_2_ support and the Pt nanocatalyst. Furthermore, this approach may provide a promising catalytic platform for fuel cell technology and a variety of applications.

## Introduction

Low-temperature fuel cell systems have been attracted more and more attention as a promising green power technology to overcome environmental and energy issues in the 21^st^ century. Compared to hydrogen-oxidation fuel cells, direct alcohol fuel cells (DAFCs) exhibited many advantages; namely, high conversion efficiency, high power density, and readily storage as well as facile transportation^1^. Up to now, carbon-supported Pt catalysts have widely utilized for both anode and cathode^2^, however, electrochemical corrosion of the carbon support^3^ causes the dissolution/detachment, Ostwald ripening, aggregation^4,5^ of the Pt nanocatalysts and thus the fuel cell performance is drastically deteriorated in long-term operation. In view of the above issues, numerous efforts have been devoted to designing non-carbon catalyst supports, which possess high corrosion resistance and strong interplay with the Pt nanocatalyst. Recently, M-doped TiO_2_ materials have emerged as a robust catalyst support in fuel cell applications owing to the synergistic effects with platinum catalyst leading to the improvement of both electrocatalytic stability and activity of the Pt-based electrocatalysts^2,6–10^.

At this juncture, the Pt nanoparticles (zero-dimensional) structures were commonly utilized in fuel cell systems^11^, however, the zero-dimensional possess some restrictions; namely, a high number of low coordination atoms and surface defects^11,12^ that directly affect their electrocatalytic activity and durability. To date, tuning the morphology and structure of the Pt catalyst has been proven to be a forward-looking approach to enhance both the activity and durability of the electrocatalyst^5,13,14^. Compared with the zero-dimensional structures, the one-dimensional structure like nanowires possessed many advantages; namely, the high surface-area-to-volume ratio, low number of surface defects, smooth single-crystalline and ability to prevent the particle agglomeration and coalescence of the Pt nanocatalysts^11,13,14^, resulting in good sensitivity and activity of the Pt-based electrocatalyst.

To be the best of our knowledge, there are a limited number of researches on the coherency of the non-carbon catalyst support and one-dimensional Pt nanowires to develop a robust electrocatalyst towards alcohol electro-oxidation reaction. In this work, we demonstrated the Pt NWs/Ti_0.7_W_0.3_O_2_ catalyst toward methanol electro-oxidation and ethanol electro-oxidation which was successfully fabricated via the simple chemical reduction route at room temperature, only using formic acid (HCOOH) as reducing agents. These observational results indicated that the Pt NWs/Ti_0.7_W_0.3_O_2_ is promising anodic catalysts for methanol electro-oxidation reaction (MOR) and ethanol electro-oxidation reaction (EOR), which can alter the conventional Pt NPs/C electrocatalysts. For instance, the robust Pt NWs/Ti_0.7_W_0.3_O_2_ electrocatalyst exhibited the lower onset potential (~0.1 V vs. NHE for MOR and ~0.2 V vs. NHE for EOR), higher mass activity (~355.29 mA/mg_Pt_ for MOR and ~325.01 mA/mg_Pt_ for EOR) and higher I_f_/I_b_ ratio (~2.70 for MOR and ~1.35 for EOR) as well as much higher electrochemical stability relative to the Pt NPs/C catalyst. The high mass activity and superior stability of the robust Pt NWs/Ti_0.7_W_0.3_O_2_ electrocatalyst could be derived from combining the merits of the one-dimensional Pt nanostructures and the mesoporous Ti_0.7_W_0.3_O_2_ catalyst support, as well as the synergistic effect between the mesoporous Ti_0.7_W_0.3_O_2_ catalyst support to the Pt nanoforms. Finally, this research can provide robust catalysts platforms for fuel cell technologies and other applications such as solar cells, water splitting.

## Results

### Characterization of the Pt NWs/Ti_0.7_W_0.3_O_2_ electrocatalysts

**Figure 1:**
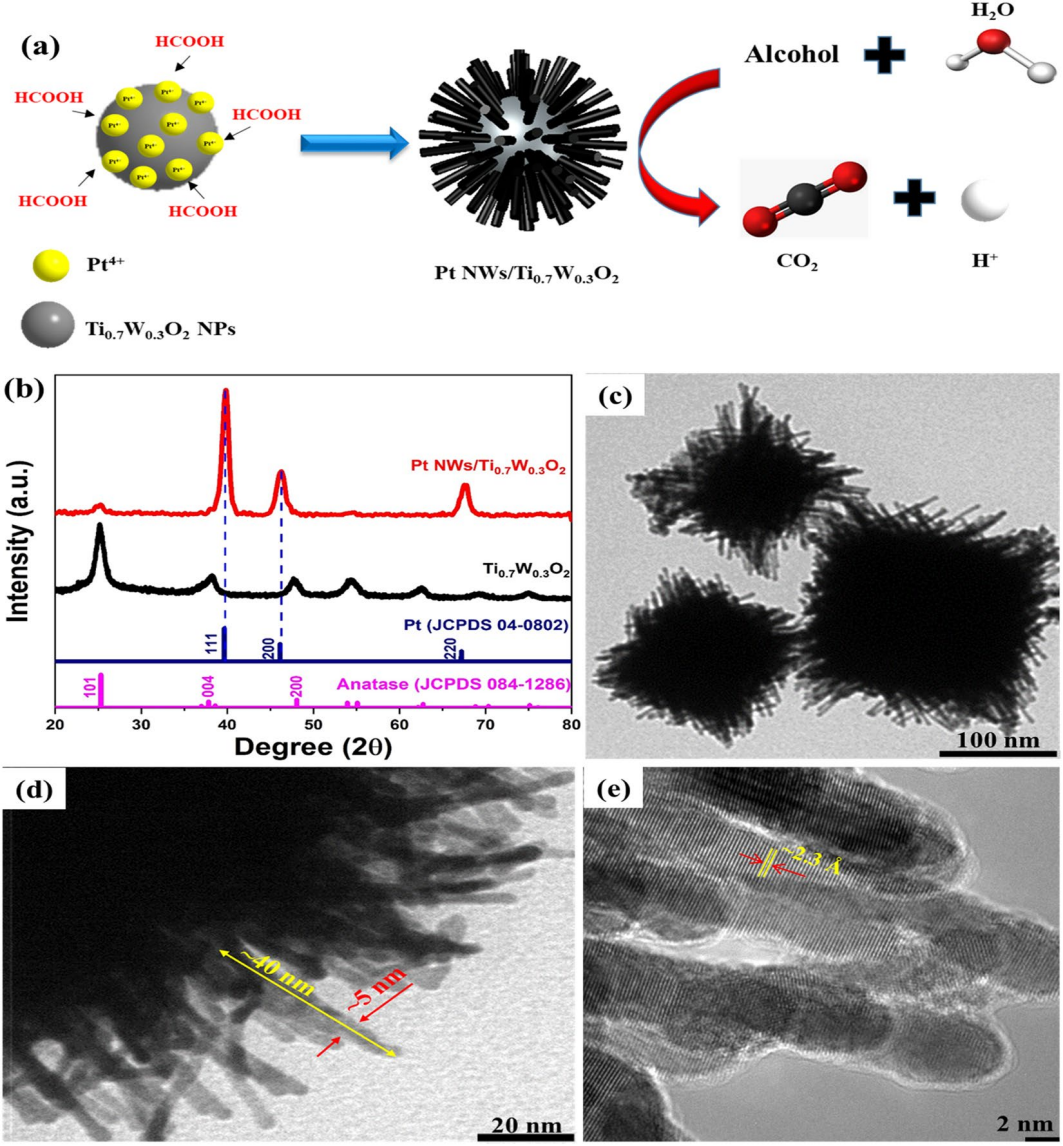
(**a**) Schematic illustration of the fabrication of the Pt NWs/Ti_0.7_W_0.3_O_2_ catalyst; (**b**) the XRD profile, (**c**,**d**) TEM images and (**e**) HR-TEM image of the Pt NWs/Ti_0.7_W_0.3_O_2_ electrocatalyst.

The mesoporous Ti_0.7_W_0.3_O_2_ support was prepared via the one-pot solvothermal route without employing surfactant/stabilizer or further heat treatment^8^ (see Figs S1–S6, Supplementary Information). In this work, the one-dimensional (1D) Pt nanocatalysts were directly grown on the mesoporous Ti_0.7_W_0.3_O_2_ support via a facile and simple chemical reduction approach at room temperature, only utilizing formic acid (HCOOH) as reducing agents (Fig. 1a). The formed structure lattice of platinum nanowires (NWs) over the Ti_0.7_W_0.3_O_2_ support was investigated by means of X-ray diffraction (XRD) measurement. As can be seen in Fig. 1b, three typical diffraction peaks of face-centered cubic (fcc) structure (JDCPS 04-0802) of the platinum metal were clearly observed at 39.76°; 46.24° and 67.45° with respect to the crystal (111), (200) and (220) facets. Importantly, the strongest peak of platinum metal was located at 39.76° correspond to crystal (111) facets, which implied that the platinum nanocatalysts were formed along the (111) direction. Interestingly, no signal of the segregation of tungsten and titanium dioxide (TiO_2_) was detected in the XRD pattern (Fig. 1b), suggesting that the mesoporous Ti_0.7_W_0.3_O_2_ catalyst support possessed the highly stable structure in reduction media with the long reaction time. Furthermore, the transmission electron microscopy (TEM) was implemented to investigate the morphology of the Pt nanoforms on the mesoporous Ti_0.7_W_0.3_O_2_ support. Figure 1c,d shows the morphology of Pt nanocatalyst to be the wire-like shape with the length ~40 nm and ~5 nm in diameter. The disuniform size of particles could be interpreted due to the agglomeration phenomena when growing the PtNWs on the surface of the Ti_0.7_W_0.3_O_2_ supports. Besides, TEM images (Fig. S7, Supplementary Information**)** exhibited the catalyst morphology to be the rhombus and sphere that could be explained due to the agglomeration of support materials and one-dimensional (1D) Pt nanocatalyst which maybe covers overall the surface of Ti_0.7_W_0.3_O_2_ support due to the high Pt loading (50 wt%) on the support. Moreover, HR-TEM image (see Fig. 1e) exhibited the fringe with a lattice spacing of ~2.3 Å corresponding to the (111) crystal plane of fcc Pt, confirming the oriented formation of Pt toward (111) facets on the surface of the supports. The mechanism of the growth of the Pt nanowires (NWs) on the Ti_0.7_W_0.3_O_2_ support could occur in a similar manner to that of Pt NWs on carbon spheres, CNT or other supports that reported in previous works^14–19^. Typically, Pt nuclei are deposited on the surface of support during the reduction of H_2_PtCl_6_ by HCOOH. Next, the freshly formed Pt nuclei act as sites for further nucleation via the continual absorption and reduction of Pt (IV) ions resulting in the formation of clustered particles. The very low reduction rate of the formic acid (HCOOH) at room temperature, which environmental favors for the anisotropic development of platinum nuclei along the (111) direction^15,16^. From this mechanism, Pt nuclei are deposited on the surface of Ti_0.7_W_0.3_O_2_ support during the reduction of H_2_PtCl_6_ by HCOOH to form Pt nuclei that act as sites for further nucleation via the continual absorption and reduction of Pt (IV) ions resulting in the formation of clustered particles and form the Pt NWs on the surface of Ti_0.7_W_0.3_O_2_ supports under the very low reduction rate and long-time reaction (72 hours) at room temperature. These outcomes suggest that the simple chemical reduction route using formic acid is a suitable approach to design the Pt NWs/Ti_0.7_W_0.3_O_2_ electrocatalyst.

**Figure 2:**
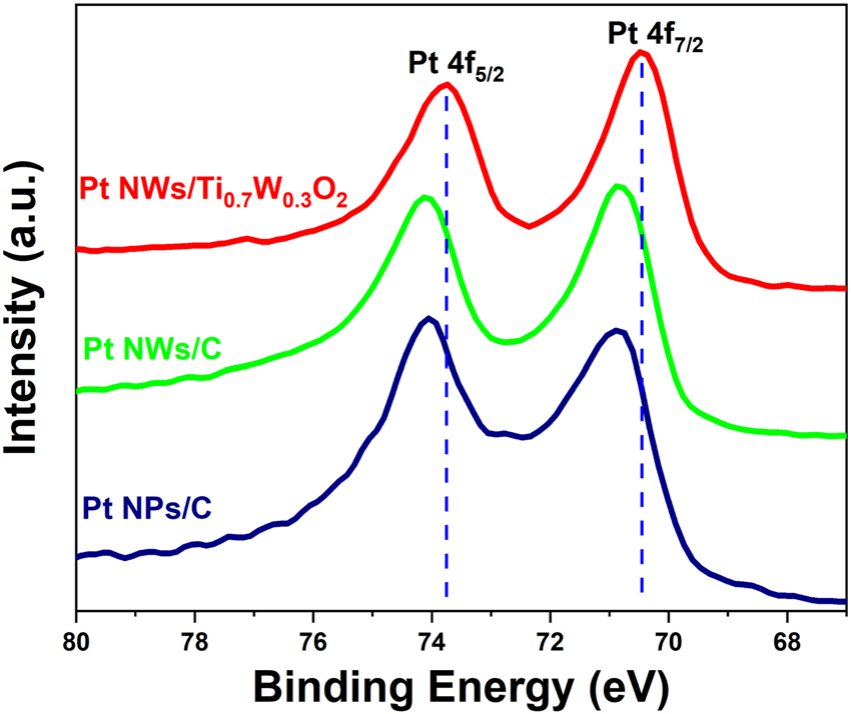
High-resolution Pt 4f spectrum of the Pt NWs/Ti_0.7_W_0.3_O_2_, Pt NWs/C and Pt NPs/C electrocatalyst.

In order to further investigate the surface characterization of the as-prepared Pt NWs/Ti_0.7_W_0.3_O_2_, Pt NWs/C and Pt NPs/C electrocatalysts, the X-ray photoelectron spectroscopy (XPS) measurement was performed. These XPS results (see Fig. 2) indicated that the Pt 4f_5/2_ and Pt 4f_7/2_ peaks of the Pt NWs/C and Pt NPs/C electrocatalyst were located at 74.08 eV and 70.80 eV, respectively, which could be which assigned to zero-valent of Pt^20,21^. Interestingly, the Pt NWs/Ti_0.7_W_0.3_O_2_ exhibited the Pt 4f_5/2_ and Pt 4f_7/2_ at 73.75 eV and 70.47 eV, respectively. It means that the negative shift to the low binding energy of Pt 4 f_5/2_ and Pt 4f_7/2_ in the Pt NWs/Ti_0.7_W_0.3_O_2_ catalyst are ascribable to the electronic transfer from Ti_0.7_W_0.3_O_2_ support to the Pt catalysts that carbon support can not exhibit the mechanism^2,6,10,22^. This results in the downshift d-band center of Pt nanocatalyst^6,22^, which normally found out in the conventional Pt-M alloy implying that the mesoporous Ti_0.7_W_0.3_O_2_ catalyst support could play a key role as co-catalyst for the Pt metal that a simple carbon support cannot^6^. The downshift d-band of the Pt nanocatalyst leading to the weak accumulation of absorbed carbonaceous species on platinum catalysts’ active sites^2,6^ and therefore can significantly enhance the electrocatalytic activity and stability of the Pt NWs/Ti_0.7_W_0.3_O_2_ electrocatalyst versus that of the conventional Pt NPs/C catalyst.

### Application of the Pt NWs/Ti_0.7_W_0.3_O_2_ towards methanol electro-oxidation reaction (MOR)

**Figure 3:**
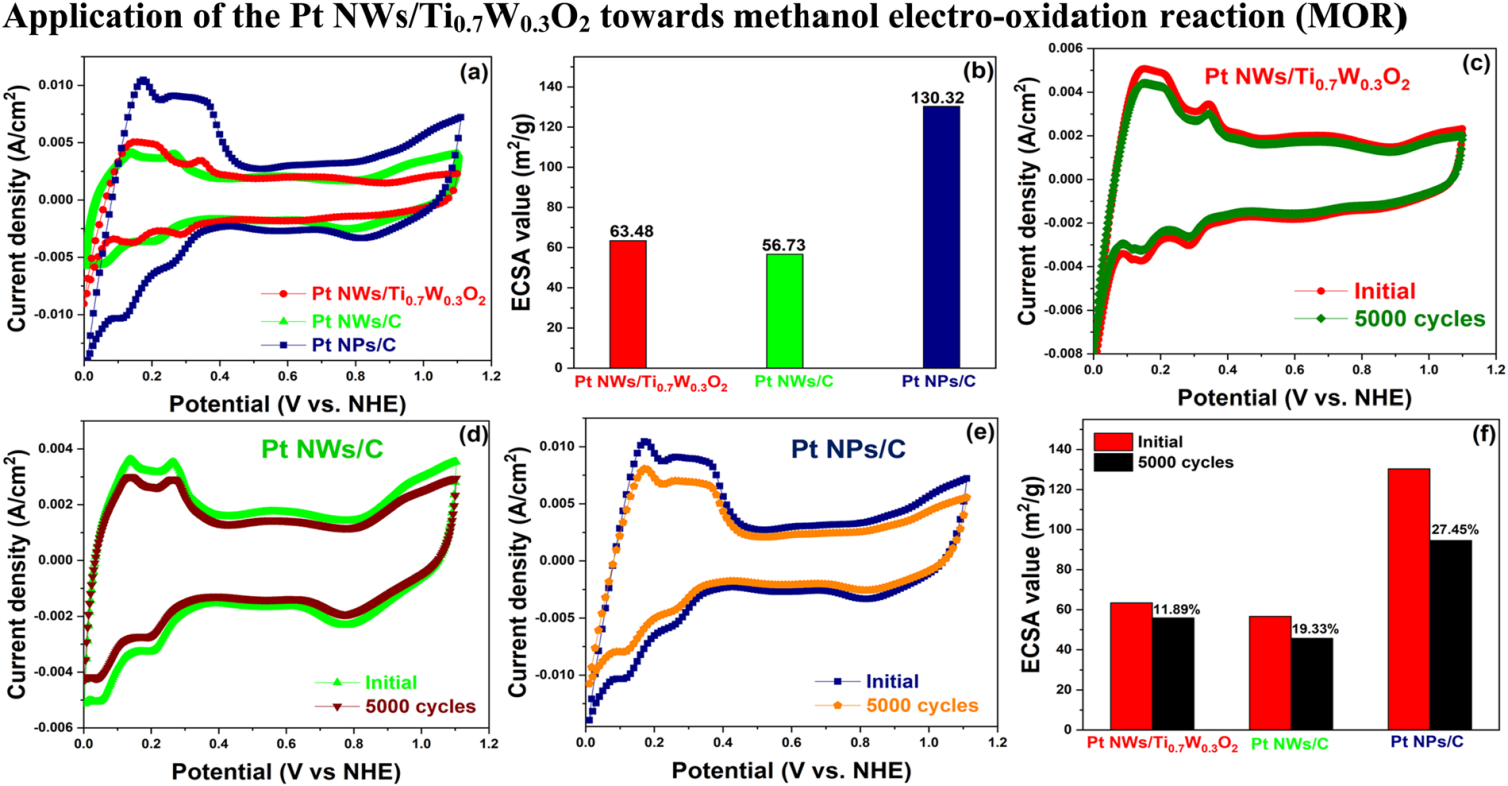
CV curves of catalysts (**a**) in N_2_-purged 0.5 M H_2_SO_4_ solution; (**b**) the ECSA value; and (**c**–**f**) after 5000 cycling test in N_2_-purged 0.5 M H_2_SO_4_ solution at a scan rate of 50 mV/s.

The electrochemical properties of the Pt NWs/Ti_0.7_W_0.3_O_2_ electrocatalysts were investigated and compared to the Pt NWs/C and the traditional Pt NPs/C in N_2_-purged 0.5 M H_2_SO_4_ aqueous solution via the cyclic voltammetry measurements. As can be seen in Fig. 3a, these catalysts show the multiple peaks in the hydrogen adsorption/desorption regions, implying that the high crystallinity of these electrocatalysts^11,14,18^. The electrochemical surface area (ECSA) of the Pt NWs/Ti_0.7_W_0.3_O_2_, Pt NWs/C, and conventional Pt NPs/C electrocatalysts, calculated from the charge of hydrogen adsorption, are around 63.48 m^2^/g; 56.73 m^2^/g, respectively, which is approximate half that of the conventional Pt/C electrocatalyst (~130.32 m^2^/g) (Fig. 3b). The low ECSA values of the Pt NWs/Ti_0.7_W_0.3_O_2_ and the Pt NWs/C catalysts versus that of the Pt NPs/C could be accounted for the reducing boundaries of the 1D morphology of the nanowires relative to the 0D morphology of the nanoparticles^23^. Besides, the accelerated durability test (ADT) in N_2_-purged 0.5 M H_2_SO_4_ at a scan rate of 50 mV/s was also employed to investigate the electrochemical stability of as-prepared electrocatalysts. After the 5000 cycling test, the ECSA loss of the Pt NWs/Ti_0.7_W_0.3_O_2_ catalysts was estimated to be 11.89% of initial ECSA value, meanwhile, the ECSA value of Pt NWs/C and conventional Pt NPs/C was significantly degraded to be ~19.33% and ~27.45% of initial ECSA value, respectively (Fig. 3c–e). The enhanced stability of the Pt NWs/Ti_0.7_W_0.3_O_2_ catalyst could be ascribed to the inherent structural and chemical durability and the superior corrosion resistance of the TiO_2_-based oxide in acidic and oxidative environments^6^.

**Figure 4:**
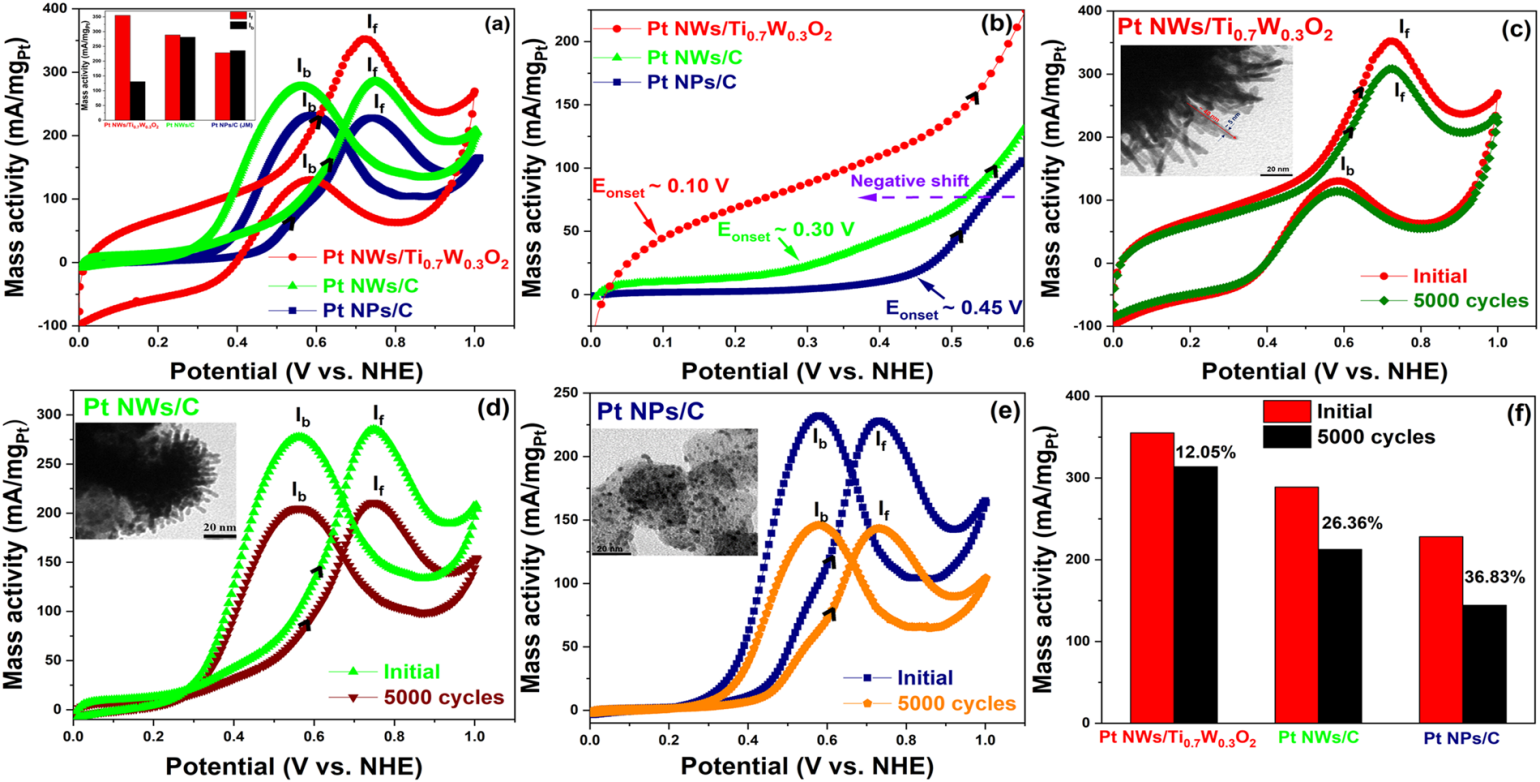
(**a**) CV curves of catalysts; inset: I_f_ and I_b_ values; (**b**) the onset potential; and (**c**–**f**) after 5000 cycling test in N_2_-purged 10 v/v % CH_3_OH/0.5 M H_2_SO_4_ solution at a scan rate of 50 mV/s.

The cyclic voltammetry measurement was carried out in N_2_-purged 10 v/v % CH_3_OH/0.5 M H_2_SO_4_ at a scan rate of 50 mV/s to evaluate the electrocatalytic activity towards methanol electro-oxidation reaction (MOR) of the Pt NWs/Ti_0.7_W_0.3_O_2_ catalyst. Figure 4a compares the CV curves of the Pt NWs/Ti_0.7_W_0.3_O_2_, the Pt NWs/C and the conventional Pt NPs/C electrocatalysts. Compared with other as-obtained electrocatalysts, the Pt NWs/Ti_0.7_W_0.3_O_2_ catalyst exhibited the highest mass activity (355.29 mA/mg_Pt_), which is ~1.23-fold and ~1.57-times higher than those of the Pt NWs/C (288.79 mA/mg_Pt_) and conventional Pt NPs/C (226.40 mA/mg_Pt_) catalysts, respectively, albeit it’s the low electrochemical surface area (ECSA) value. The great electrocatalytic activity of the Pt NWs/Ti_0.7_W_0.3_O_2_ catalysts is ascribable to the formed Pt nanoforms along the (111) orientation, which possessed the most activity towards methanol electro-oxidation owing to the low poisoning rate^24^. Furthermore, the potential at which the methanol oxidation starts (i.e., the onset potential, E_onset_) of the Pt NWs/Ti_0.7_W_0.3_O_2_ catalyst was found to be the lowest (~0.1 V vs. NHE), which is negatively shifted about 200 mV and 350 mV with respect to the Pt NWs/C (~0.3 V vs. NHE) and the conventional Pt NPs/C (~0.45 V vs. NHE) (see Fig. 4b), implying that the methanol electro-oxidation reaction (MOR) on the Pt NWs/Ti_0.7_W_0.3_O_2_ catalyst was performed easier and faster than the Pt NWs/C and Pt NPs/C electrocatalyst. Consequently, the ratio of the forward peak current density (I_f_) and a negative-going current density (I_b_) is generally represented the resistance to the poisoning of the accumulation of carbonaceous species^2,25^. Interestingly, the Pt NWs/Ti_0.7_W_0.3_O_2_ catalyst exhibited the highest I_f_/I_b_ values in comparison with the Pt NWs/C and the conventional Pt NPs/C electrocatalyst. The I_f_/I_b_ ratio of three different catalysts are shown in order: Pt NWs/Ti_0.7_W_0.3_O_2_ (~2.70) > Pt NWs/C (~1.03) > Pt NPs/C (0.97), suggesting that the Pt NWs/Ti_0.7_W_0.3_O_2_ electrocatalysts possessed the best CO-tolerance in comparison with the Pt NWs/C and the conventional Pt NPs/C catalysts towards MOR. The high I_f_/I_b_ value of the Pt NWs/Ti_0.7_W_0.3_O_2_ electrocatalyst compared to that of the Pt NWs/C electrocatalyst could be explained due to the strong interaction between Pt nanocatalyst and Ti_0.7_W_0.3_O_2_ support, resulting in making weak adsorption of CO-like species on the sites of Pt nanocatalyst^2,6,26^ (Table 1). For the Pt NPs/C electrocatalyst, the high I_f_/I_b_ value of the Pt NWs/Ti_0.7_W_0.3_O_2_ electrocatalyst was attributable to the strong interaction between Pt nanocatalyst and Ti_0.7_W_0.3_O_2_ support as well as the advantages of one-dimensional (1D) structure of Pt nanocatalyst such as (i) long segments of smooth crystal planes, (ii) a low number of surface defects, leading to good sensitivity and and activity for methanol electro-oxidation reaction^11,18^ and (iii) unique one-dimensional (1D) Pt morphology, resulting in improving mass transport and electron transfers during electrocatalytic reactions^11,17,18,27,28^.

In order to examine the durability of the Pt NWs/Ti_0.7_W_0.3_O_2_ catalyst towards MOR, the 5000 potential cycling measurement was conducted in N_2_-purged 10 v/v % CH_3_OH/0.5 M H_2_SO_4_ solution at a scan rate of 50 mV/s. Figure 4c–e show the CVs of three different electrocatalysts before and after the test. As results indicated that the Pt NWs/Ti_0.7_W_0.3_O_2_ electrocatalysts exhibited superior stability in comparison with the Pt NWs/C, and the conventional Pt NPs/C catalysts. Particularly, the Pt NWs/Ti_0.7_W_0.3_O_2_ catalyst demonstrated the deterioration of the mass activity to be around 12.05% of the initial mass activity, which was ~2.18-times and ~3.06-fold lower than those of the Pt NWs/C (~26.36%), and the conventional Pt NPs/C electrocatalysts (~36.83%), respectively (see Fig. 4f). It can be concluded that the stability of the Pt NWs/Ti_0.7_W_0.3_O_2_ was greatly enhanced towards the MOR.

### Application of the Pt NWs/Ti_0.7_W_0.3_O_2_ towards ethanol electro-oxidation reaction (EOR)

**Figure 5:**
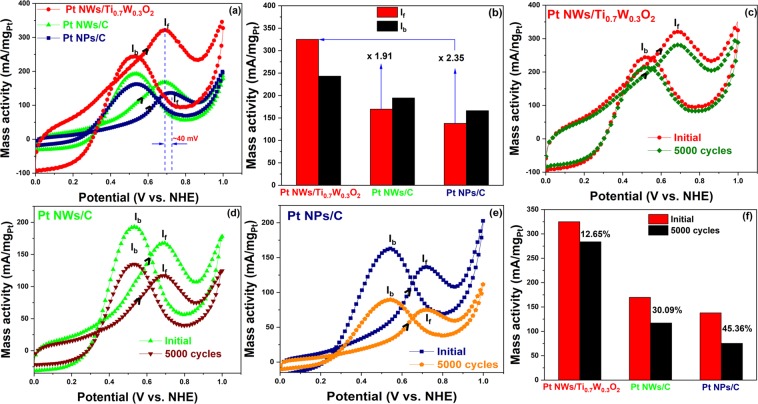
(**a**) CV curves; (**b**) I_f_ and I_b_ values and (**c**–**f**) after 5000 cycling test of catalysts in N_2_-purged 10 v/v % C_2_H_5_OH/0.5 M H_2_SO_4_ solution at a scan rate of 50 mV/s.

Until now, ethanol has emerged as green fuel sources, which can alter for methanol because of lower toxicity and market cost, however, one of the most major challenges is the development of the electrocatalyst with the great electrocatalytic stability and activity towards ethanol electro-oxidation reaction (EOR)^11^. With the unique electrocatalytic properties towards MOR, we also further evaluated the catalytic activity and stability of the Pt NWs/Ti_0.7_W_0.3_O_2_ electrocatalyst for the EOR. Figure 5a shows the CV curves of three different electrocatalysts in N_2_-purged 10 v/v % C_2_H_5_OH/0.5 M H_2_SO_4_ solution at a scan rate of 50 mV/s. By comparing the positive-going and negative-going EOR waves in terms of the peak potential and peak current, the Pt NWs anchored over the mesoporous Ti_0.7_W_0.3_O_2_ catalyst support exhibited the higher electrocatalytic activity than the Pt NWs/C and the conventional Pt NPs/C electrocatalyst. On the positive-going sweep, both the onset potential and ethanol electro-oxidation potential of the Pt NWs/Ti_0.7_W_0.3_O_2_ catalyst were negatively shifted compared to those of the conventional Pt NPs/C to be ~300 mV and ~40 mV, respectively (see Fig. 5a), implying the better CO-tolerance of the as-prepared Pt NWs/Ti_0.7_W_0.3_O_2_ catalyst due to the facile removal of the adsorbed carbonaceous intermediate species^14,29^. Moreover, the mass activity of the Pt NWs/Ti_0.7_W_0.3_O_2_ catalyst was found to be around 325.01 mA/mg_Pt_, which was ~1.91-fold and ~2.35-times higher than those of the Pt NWs/C (~169.73 mA/mg_Pt_), and the traditional Pt NPs/C (~137.98 mA/mg_Pt_), respectively (Fig. 5b), suggesting that the catalytic activity towards ethanol electro-oxidation reaction (EOR) of the Pt NWs/Ti_0.7_W_0.3_O_2_ catalyst was drastically improved. The great mass activity of the as-obtained Pt NWs/Ti_0.7_W_0.3_O_2_ catalyst could result from the strong interaction between the Pt nanocatalyst and Ti_0.7_W_0.3_O_2_ support which provides active species for catalytic reaction resulting in enhancing the dehydrogenation of ethanol^30–33^. For the negative-going waves, the anodic peak corresponds to the continuous oxidation of ethanol when the adsorbed intermediate species on the catalyst’s surface in the forward sweep are removed^34^. Importantly, the Pt NWs/Ti_0.7_W_0.3_O_2_ catalyst possessed the highest I_f_/I_b_ values, which showed in order: Pt NWs/Ti_0.7_W_0.3_O_2_ (~1.35) > Pt NWs/C (~0.87) > Pt NPs/C (~0.83), suggesting that the Pt NWs/Ti_0.7_W_0.3_O_2_ possessed the high resistance to poisoning of the accumulation of carbonaceous species (Table 2). Besides the advantages of the one-dimensional Pt morphology, the electronic transfer from the mesoporous Ti_0.7_W_0.3_O_2_ support to the Pt nanocatalyst (so-called “electronic effect”) making the downshift d-band center of the Pt nanocatalyst and lower the adsorption energy of CO_ads_, facilitating the oxidation of CO_ads_ at a lower potentials^2,24^, resulting in the significant enhancement of both the electrocatalytic activity and stability of the Pt NWs/Ti_0.7_W_0.3_O_2_ catalyst.

**Table 1:** Electrochemical characterization towards MOR of the Pt-based NWs catalyst.

Catalysts	ECSA (m^2^/g_Pt_)	Onset potential (V)	Mass activity (mA/mg_Pt_)	I_f_/I_b_	Ref
Pt NWs/Ti_0.7_W_0.3_O_2_	63.48	0.10 V vs. NHE	355.29	2.70	This work
Pt NWs/C	56.73	0.30 V vs. NHE	288.79	1.03	This work
Pt NPs/C	130.32	0.45 V vs. NHE	226.40	0.97	This work
Pt NWs/Ti_0.7_Ru_0.3_O_2_	21.05	0.32 V vs. NHE	—	1.23	^14^
BPt NW/RGO	25.90	0.40 V vs. Ag/AgCl	350.00	1.01	^35^
Mesoporous Pt NWs	40.50	0.50 V vs. NHE	398.00	1.15	^34^
Mesoporous Pt NWs	40.20	0.39 V vs. Ag/AgCl	192.80	1.32	^36^
Pt NWs/CS	55.60	0.1 V vs. SCE	450.00	1.20	^37^
Commercial Pt/C	43.30	0.3 V vs. SCE	194.00	0.80	^37^

**Table 2:** Electrochemical characterization towards EOR of the Pt-based NWs catalyst.

Catalysts	ECSA (m^2^/g_Pt_)	Onset potential (V)	Mass activity (mA/mg_Pt_)	I_f_/I_b_	Ref
Pt NWs/Ti_0.7_W_0.3_O_2_	63.48	0.20 V vs. NHE	325.01	1.35	This work
Pt NWs/C	56.73	0.42 V vs. NHE	169.73	0.87	This work
Pt NPs/C	130.32	0.50 V vs. NHE	137.98	0.83	This work
Pt/TiO_2_-C	18.47	—	261.30	—	^38^
Pt-Mo-Ni NWs	—	0.10 V vs. SCE	865.80	0.62	^39^
PdPt@PtPd CSNDs	27.40	0.50 V vs. RHE	45.00	1.01	^40^
PtSn/Fe-C (1:1)	57.80	0.10 V vs. NHE	—	0.80	^41^
Pt_0.7_Rh_0.3_/C_50_(SnO_2_:Sb)_75_	—	0.57 V vs. RHE	—	1.02	^11^
Pt NWs/CS	55.60	0.10 V vs. SCE	278.00	1.10	^37^
Cothatmmercial Pt/C	43.30	0.40 V vs. NHE	204.00	0.70	^37^
PtSn/XC-72R	—	0.20 V vs. SCE	764.10	0.85	^42^

The electrocatalytic stability of the Pt NWs/Ti_0.7_W_0.3_O_2_ towards EOR was further investigated via the accelerated durability test (ADT) in N_2_-purged 10 v/v % C_2_H_5_OH/0.5 M H_2_SO_4_ aqueous solution. These outcomes indicated that the Pt NWs/Ti_0.7_W_0.3_O_2_ catalyst possessed the superior stability towards ethanol electro-oxidation in comparison with the Pt NWs/C and the conventional Pt NPs/C electrocatalysts. For instance, after 5000 cycling test, the mass activity of the Pt NWs/Ti_0.7_W_0.3_O_2_ electrocatalyst was found to be around 283.89 mA/mg_Pt_ with respect to the deterioration to be ~12.65% of the initial mass activity (~325.01 mA/mg_Pt_), meanwhile, the Pt NWs/C and conventional Pt NPs/C electrocatalysts showed the decay to be around ~30.09% (from 169.73 mA/mg_Pt_ dropped to 118.66 mA/mg_Pt_) and ~45.36% (from 137.98 mA/mg_Pt_ to 75.41 mA/mg_Pt_), respectively (see Fig. 5c–f). The significant degradation of the Pt NWs/C and the conventional Pt NPs/C electrocatalyst is attributable to the poor durability of the carbon-based support resulting in the detachment/dissolution, Ostwald ripening of the Pt nanocatalyst^2^.

**Figure 6:**
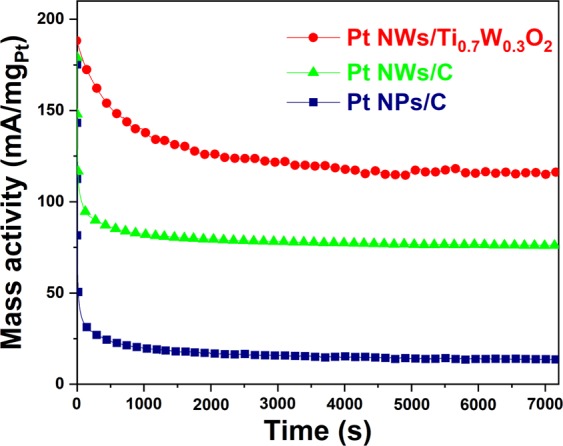
Chronoamperogams of the as-obtained electrocatalysts in N_2_-purged 10 v/v % C_2_H_5_OH/0.5 M H_2_SO_4_ solution at the oxidation potential of 0.7 V for 7200 s.

The chronoamperometry measurement in N_2_-purged 10 v/v % C_2_H_5_OH/0.5 M H_2_SO_4_ aqueous solution at the immobilized potential of 0.7 V for 7200 s was carried out to investigate the electrocatalytic stability of the Pt NWs/Ti_0.7_W_0.3_O_2_ electrocatalyst towards EOR. As can be seen in Fig. 6, the Pt NWs/Ti_0.7_W_0.3_O_2_ catalyst exhibited the initial mass activity to be 187.04 mA/mg_Pt_, which is higher than those of the Pt NWs/C (~178.99 mA/mg_Pt_) and the conventional Pt NPs/C (~175.21 mA/mg_Pt_). After 7200 s test, the mass activity of the Pt NWs/Ti_0.7_W_0.3_O_2_ electrocatalyst was remained to be around 116.80 mA/mg_Pt_, which is ~1.54-fold and ~8.44-fold higher than those of the Pt NWs/C (75.90 mA/mg_Pt_) and the Pt NPs/C (13.84 mA/mg_Pt_) at the same time, respectively. The decay rate of the as-prepared catalysts is showed in order: Pt NWs/Ti_0.7_W_0.3_O_2_ (~0.59 mA/mg_Pt_.min) < Pt NWs/C (~0.86 mA/cm^2^.min) < Pt NPs/C (~1.34 mA/mg_Pt_.min). The superior durability of the Pt NWs/Ti_0.7_W_0.3_O_2_ electrocatalyst relative to the conventional Pt NPs/C could be interpreted due to the lower vulnerability to dissolution, Ostwald ripening and aggregation of the 1D Pt structure (nanowires) than 0D Pt structure (nanoparticles)^11,13,14^. In addition, the high corrosion resistance of TiO_2_-based oxide in acidic and oxidative environments^14^ as well as the electronic effect between the mesoporous Ti_0.7_W_0.3_O_2_ support and the Pt nanocatalysts, which results in the weak adsorption of carbonaceous intermediate species on the active sites of the Pt nanocatalysts and, consequently, improve the catalytic stability of the Pt NWs/Ti_0.7_W_0.3_O_2_ catalyst.

## Discussion

In brief, we introduce the robust Pt NWs/Ti_0.7_W_0.3_O_2_ electrocatalyst, which was prepared via a simple chemical reduction route at room temperature without utilizing surfactant/stabilizers or template. The experimental outcomes demonstrated that the robust Pt NWs/Ti_0.7_W_0.3_O_2_ catalyst is a promising electrocatalyst towards the methanol electro-oxidation and ethanol electro-oxidation. For instance, the Pt NWs/Ti_0.7_W_0.3_O_2_ electrocatalyst exhibited the lower onset potential (~0.1 V vs. NHE for MOR and ~0.2 V vs. NHE for EOR), higher mass activity (~355.29 mA/mg_Pt_ for MOR and ~325.01 mA/mg_Pt_ for EOR), and the greater I_f_/I_b_ ratio (~2.70 for MOR and ~1.35 for EOR) along with the superior stability in acidic and oxidative environment related to the conventional Pt/C catalyst. These enhancements of the Pt NWs/T_0.7_W_0.3_O_2_ catalyst are attributable to the merits of the one-dimensional Pt structure and the mesoporous Ti_0.7_W_0.3_O_2_ catalyst support, as well as the electron transfers from the Ti_0.7_W_0.3_O_2_ catalyst support to the Pt nanowires, which was evidenced via the XPS spectroscopy, leading to the weak linkage of intermediate carbonaceous species on the active surface of the Pt nanocatalyst. Furthermore, this approach may provide a robust catalytic platform for fuel cell technologies and a variety of applications.

## Methods

### Fabrication of the Pt NWs/Ti_0.7_W_0.3_O_2_ electrocatalyst

In this work, the simple chemical reduction route was utilized to produce the Pt NWs/Ti_0.7_W_0.3_O_2_ catalyst consisted of using the chloroplatinic acid hydrate (H_2_PtCl_6_.xH_2_O) and the as-prepared Ti_0.7_W_0.3_O_2_ catalyst support as starting precursors as well as the formic acid (HCOOH) as reducing agent. Briefly, a mixture of 8 mL H_2_O and 0.62 mL 0.05 M H_2_PtCl_6_ aqueous solution and 1.2 mL HCOOH was stirred for 15 min to generate a homogenous solution. Next, 6 mg Ti_0.7_W_0.3_O_2_ supports were ultrasonically dissolved into the above solution for 15 min to create a homogeneous suspension. Afterward, the as-prepared suspension was stored at room temperature for 72 hours to fabricate the 50 wt % Pt NWs/Ti_0.7_W_0.3_O_2_ catalyst. Finally, the obtained product was rinsed copiously with purified water and then dried at 80 °C overnight for further analysis. For comparison, the 50 wt % Pt NWs were grown on Vulcan XC-72 support at the same condition.

### Material characterization

The structure information of the formed Pt nanowires over the mesoporous Ti_0.7_W_0.3_O_2_ support was measured via the X-ray diffraction (XRD) measurement operated on a D2 PHASER-Brucker using Cu K_α_ radiation at 30 kV. The transmission electron microscopy (TEM) measurement was conducted on the JEOL-LEM 1400 microscope at an accelerating voltage of 3800 V to examine the morphology of the as-prepared Pt NWs/Ti_0.7_W_0.3_O_2_ catalyst. Furthermore, the X-ray photoelectron spectroscopy (XPS) was implemented to investigate the surface properties of the as-obtained Pt NWs/Ti_0.7_W_0.3_O_2_ electrocatalyst.

### Electrochemical properties

An EC-LAB Electrochemistry instrument (Bio-Logic SAS) with an Ag/AgCl/Sat. KCl electrode, and a Pt gauze, as well as glassy carbon electrode with 5 mm in diameter with respect to a reference electrode and the counter electrode as well as a working electrode, were used for investigating all electrochemical tests. The electrocatalytic activity towards methanol electro-oxidation reaction (MOR) and ethanol electro-oxidation reaction (EOR) of the as-obtained catalysts was recorded at a scan rate of 50 mV/s in N_2_-statured 10 v/v % CH_3_OH/0.5 M H_2_SO_4_ solution and N_2_-statured 10 v/v % C_2_H_5_OH/0.5 M H_2_SO_4_ solution, respectively. Furthermore, the ADT test was performed in the range of 0 V to 1.0 V (vs. NHE) for 5000 cycles at a scan rate of 50 mV/s at room temperature in N_2_-purged 10 v/v% CH_3_OH/0.5 M H_2_SO_4_ and in N_2_-purged 10 v/v% C_2_H_5_OH/0.5 M H_2_SO_4_ for methanol electro-oxidation reaction (MOR) and ethanol electro-oxidation reaction (EOR), respectively. All potential ranges in this work were reported with the normal hydrogen electrode (NHE) scale. The catalyst ink preparation: the catalyst powder was ultrasonicated in a solution comprising ethanol absolute and 0.5% Nafion within 30 min. Before the catalyst ink placement, the surface of the glassy carbon disk was polished with 0.5 µm BAS and then washed with ethanol as well as purified water. To start with, the catalyst electrode was activated by 100 cycles at a scan rate of 50 mV/s. In this work, the Pt loading onto the glassy carbon electrode was maintained at 0.13 mg/cm^2^ in all electrochemical tests.

## Supplementary information

Supplementary information

Supplementary information
