# Supplementary material for: Wire-like Pt on mesoporous Ti0.7W0.3O2 Nanomaterial with Compelling Electro-Activity for Effective Alcohol Electro-Oxidation
Source: Sci Rep. 2019 Oct 15;9:14791. doi: 10.1038/s41598-019-51235-4 (PMC6794307; doi:10.1038/s41598-019-51235-4)

**Graphical Abstract**

**Wire-like Pt on** **mesoporous Ti_0.7_W_0.3_O_2_ Nanomaterial with** **Compelling Electro-Activity for Effective** **Alcohol Electro-Oxidation**

Hau Quoc Pham^a,b^, Tai Thien Huynh^a,c^, Anh Tram Ngoc Mai^a^, Thang Manh Ngo^a^,

Long Giang Bach^b,*^, Van Thi Thanh Ho^c,*^

*^a^Ho Chi Minh City University of Technology, VNU-HCM*

*^b^NTT Hi-Tech Institute, Nguyen Tat Thanh University, Ho Chi Minh City, Vietnam*

*^c^Hochiminh City University of Natural Resources and Environment (HCMUMRE), Vietnam*

*^*^Corresponding author's e-mail:* [*httvan@hcmunre.edu.vn*](mailto:httvan@hcmunre.edu.vn)*,* [*blgiang@ntt.edu.vn*](mailto:blgiang@ntt.edu.vn)

**Graphical Abstract**


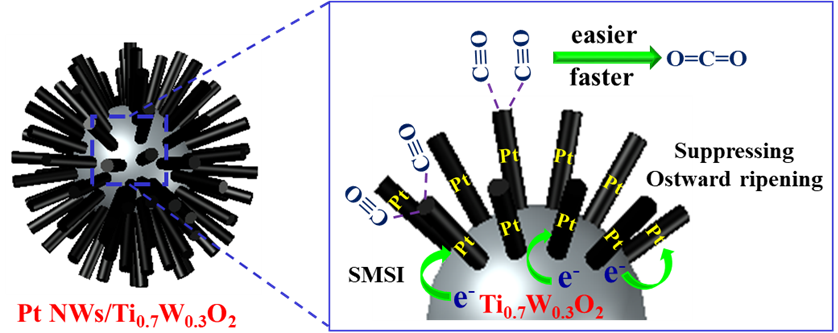

Supplement: Supplementary file 1 — Supplementary information [file 41598_2019_51235_MOESM1_ESM.docx]
