# Supplementary material for: Wire-like Pt on mesoporous Ti0.7W0.3O2 Nanomaterial with Compelling Electro-Activity for Effective Alcohol Electro-Oxidation
Source: Sci Rep. 2019 Oct 15;9:14791. doi: 10.1038/s41598-019-51235-4 (PMC6794307; doi:10.1038/s41598-019-51235-4)
Supplement: Supplementary file 2 — Supplementary information [file 41598_2019_51235_MOESM2_ESM.docx]

**Supplementary information**

**Wire-like Pt on** **mesoporous Ti_0.7_W_0.3_O_2_ Nanomaterial with** **Compelling Electro-Activity for Effective** **Alcohol Electro-Oxidation**

Hau Quoc Pham^a^, Tai Thien Huynh^a,b^, Anh Tram Ngoc Mai^a^, Thang Manh Ngo^a^,

Long Giang Bach^c,*^, Van Thi Thanh Ho^b,*^

*^a^Ho Chi Minh City University of Technology, VNU-HCM*

*^b^Hochiminh City University of Natural Resources and Environment (HCMUMRE), Vietnam*

*^c^NTT Hi-Tech Institute, Nguyen Tat Thanh University, Ho Chi Minh City, Vietnam*

*^*^Corresponding author's e-mail:* [*httvan@hcmunre.edu.vn*](mailto:httvan@hcmunre.edu.vn)*,* [*blgiang@ntt.edu.vn*](mailto:blgiang@ntt.edu.vn)

**Preparation of the mesoporous Ti_0.7_W_0.3_O_2_ support**

The mesoporous Ti_0.7_W_0.3_O_2_ catalyst support was fabricated via the facile and simple solvothermal approach, only using tungsten (VI) chloride (WCl_6_, 99.9%, Sigma-Aldrich), titanium (IV) chloride (TiCl_4_, 99.5%, Aladdin) with respect to start precursors of W and Ti and Ethanol (99.9%, Merck) as solvent, without utilizing surfactant/stabilizer or further heat treatment^1^. Firstly, 0.238 mg of WCl_6_ was dispersed into 50 mL of absolute ethanol and then 0.155 mL TiCl_4_ was added to the above solution. Next, this mixture was dropped into a Teflon-line autoclave and then transfer to an over, the reaction was performed at 200^o^C for 10 hours. Afterward, the as-obtained suspension was naturally cooled to room temperature and copiously rinsed with acetone (99.9%, Merck) and purified water. Finally, the as-prepared Ti_0.7_W_0.3_O_2_ support was dried at 80^o^C for further analysis. ^1, 2^

**Characterization of the mesoporous Ti_0.7_W_0.3_O_2_ catalyst support**

**
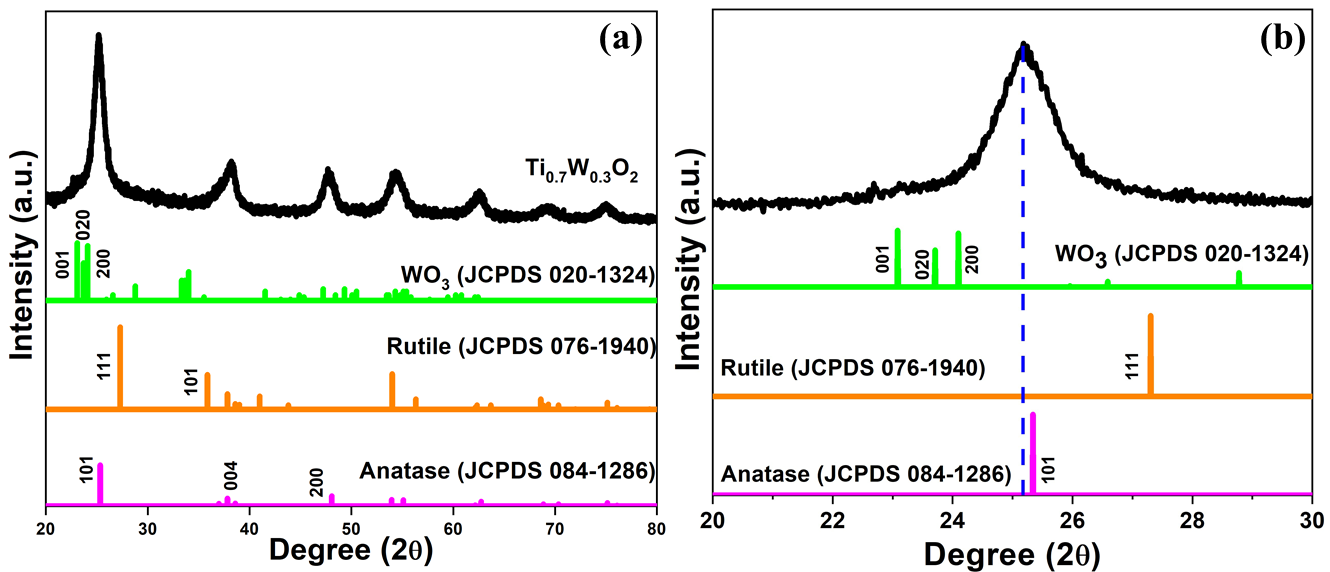
**

**Fig. S1 (**a) The XRD patterns and (b) the XRD profile in the 2θ range from 22^o^ to 30^o^ of the mesoporous Ti_0.7_W_0.3_O_2_ catalyst support

The structure information of the as-obtained Ti_0.7_W_0.3_O_2_ nanoparticles (NPs) was recorded by means of X-ray diffraction (XRD) measurement. **Fig. S1a** indicated that the as-prepared Ti_0.7_W_0.3_O_2_ NPs exhibited the anatase-TiO_2_ structure (JCPDS 084-1286) with the typical diffraction peaks at 25.3^o^; 38.1^o^; 47.5^o^; 54.4^o^ and 62.8^o^ with respect to (101); (004); (200); (105) and (204). No typical diffraction peaks of tungsten oxide (JCPDS 020-1324) or the segregation of tungsten and titanium oxide was detected in XRD profile (**Fig. S1a).** Importantly, **Fig. S1b** showed that highest diffraction peaks corresponding to the (101) crystal plane of the as-obtained Ti_0.7_W_0.3_O_2_ NPs was negatively shifted versus that of the standard anatase-TiO_2_ structure (JCPDS 084-1286). These experimental results suggested that the tungsten successfully doped into TiO_2_ lattice and therefore the formation of the solid solution with anatase-TiO_2_ structure. ^2^

**
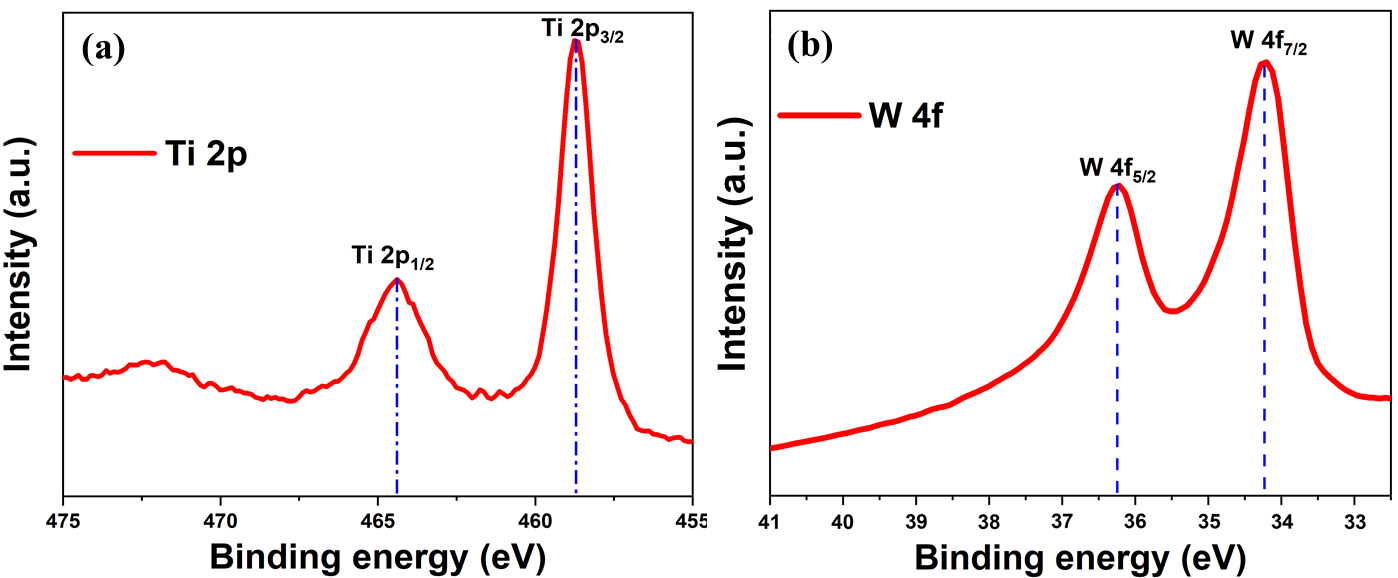
**

**Fig. S2** (a) High-resolution Ti 2p spectrum and (b) high-resolution W 4f spectrum of the mesoporous Ti_0.7_W_0.3_O_2_ catalyst support

The X-ray photoelectron spectroscopy (XPS) measurement was performed to further investigate the characterization of the as-prepared Ti_0.7_W_0.3_O_2_ NPs. As can be seen in **Fig. S2a**, the Ti 2p_1/2_ and Ti 2p_3/2_ peaks were detected at ~464.5 eV and ~458.75 eV, which was slightly negatively shifted in comparison with of the pure TiO_2_ (464.0 eV for Ti 2p_1/2_ and 458.4 eV for Ti 2p_3/2_)^3^. Furthermore, **Fig. S2b** showed the W 4f_5/2_ and W 4f_7/2_ peaks located at ~36.25 eV and ~34.25 eV, respectively, which was shifted to low binding energy in comparison with the pure WO_3_ (37.4 eV for W 4f_5/2_ and 35.3 eV for W 4f_7/2_)^4, 5^. These negative shifts could be accounted for the presence of tungsten into TiO_2_ lattice and therefore the formation of the W-O-Ti linkages^3, 4^.


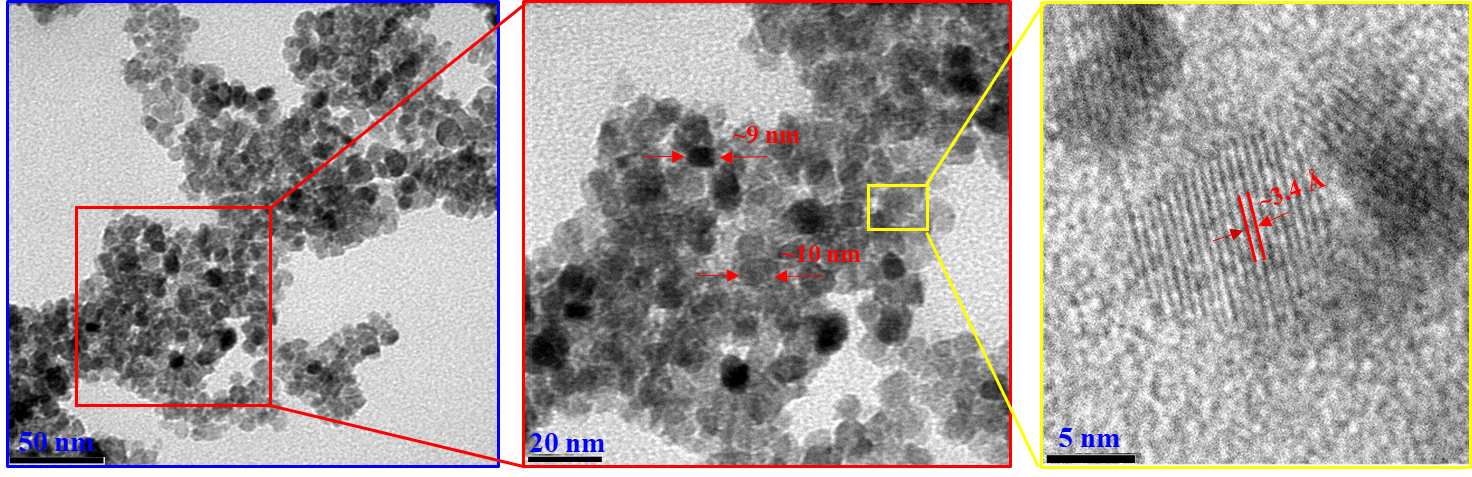

**Fig. S3** (a, b) TEM images and (c) HR-TEM image of the as-obtained Ti_0.7_W_0.3_O_2_ NPs

The particle size and morphology of the as-synthesized Ti_0.7_W_0.3_O_2_ support was recorded via the TEM and HR-TEM analysis. **Fig. S3 (a,b)** indicated that the morphology of the Ti_0.7_W_0.3_O_2_ support was found to be the spherical-like with ~9 nm in diameter. Moreover, the HR-TEM image (**Fig. S3c**) exhibited the well-defined fringes at ~3.4 Å corresponding to the spacing of the (101) crystal plane of the anatase-TiO_2_, which closely agreed with XRD patterns (**Fig. S1**).

**
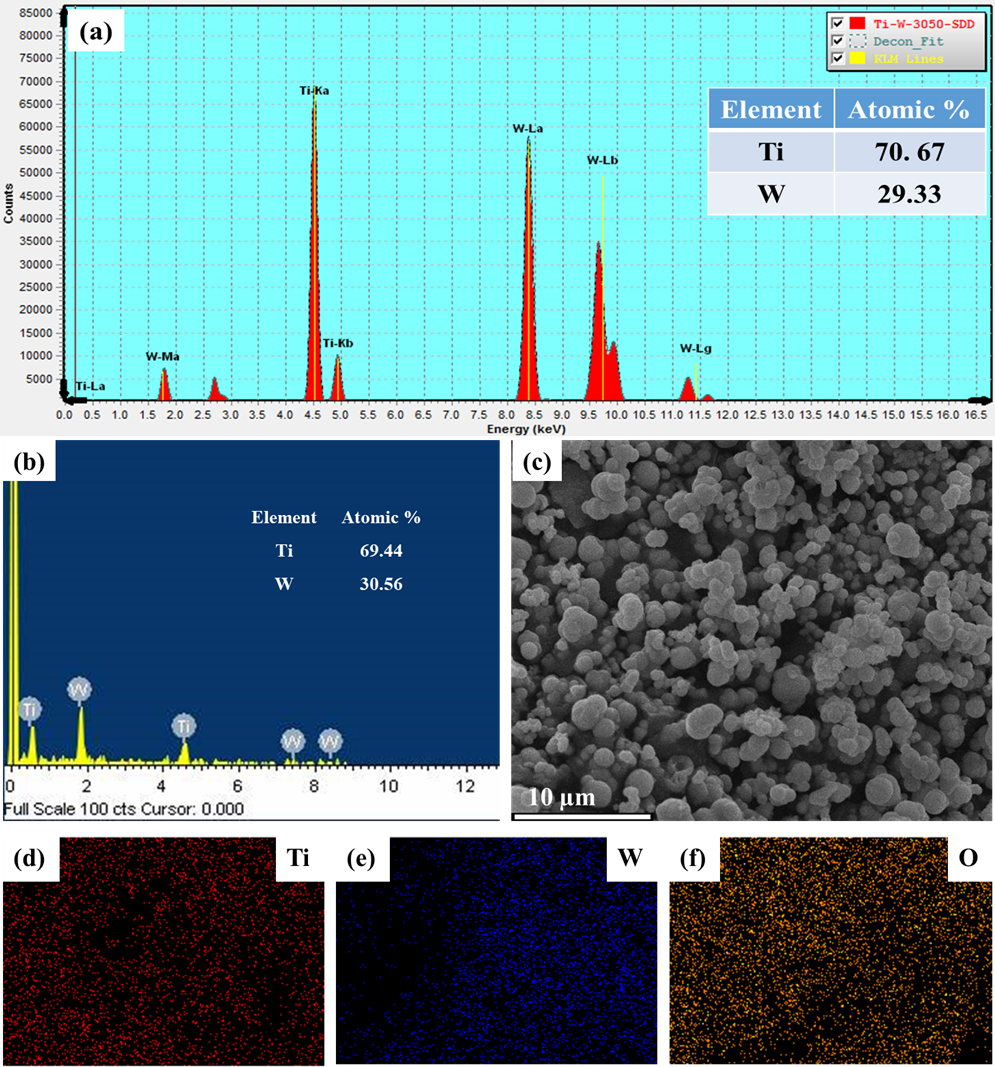
**

**Fig. S4** (a) X-ray fluorescence spectroscopy, (b-c) EDX-SEM spectroscopy and (d-f) elemental mapping of the mesoporous Ti_0.7_W_0.3_O_2_ catalyst support

To investigate the elemental composition of the as-obtained Ti_0.7_W_0.3_O_2_ support, the XRF and SEM-EDX measurements were implemented. As can be seen in **Fig. S4 (a,b)**, the element proportions of Ti and W were found to be approximately the desired elemental composition of Ti: W and 70:30. Furthermore, the elemental mapping (**Fig. S4 (d-f)**) showed the relatively uniform distribution of elements in the as-prepared Ti_0.7_W_0.3_O_2_ nanoparticles.


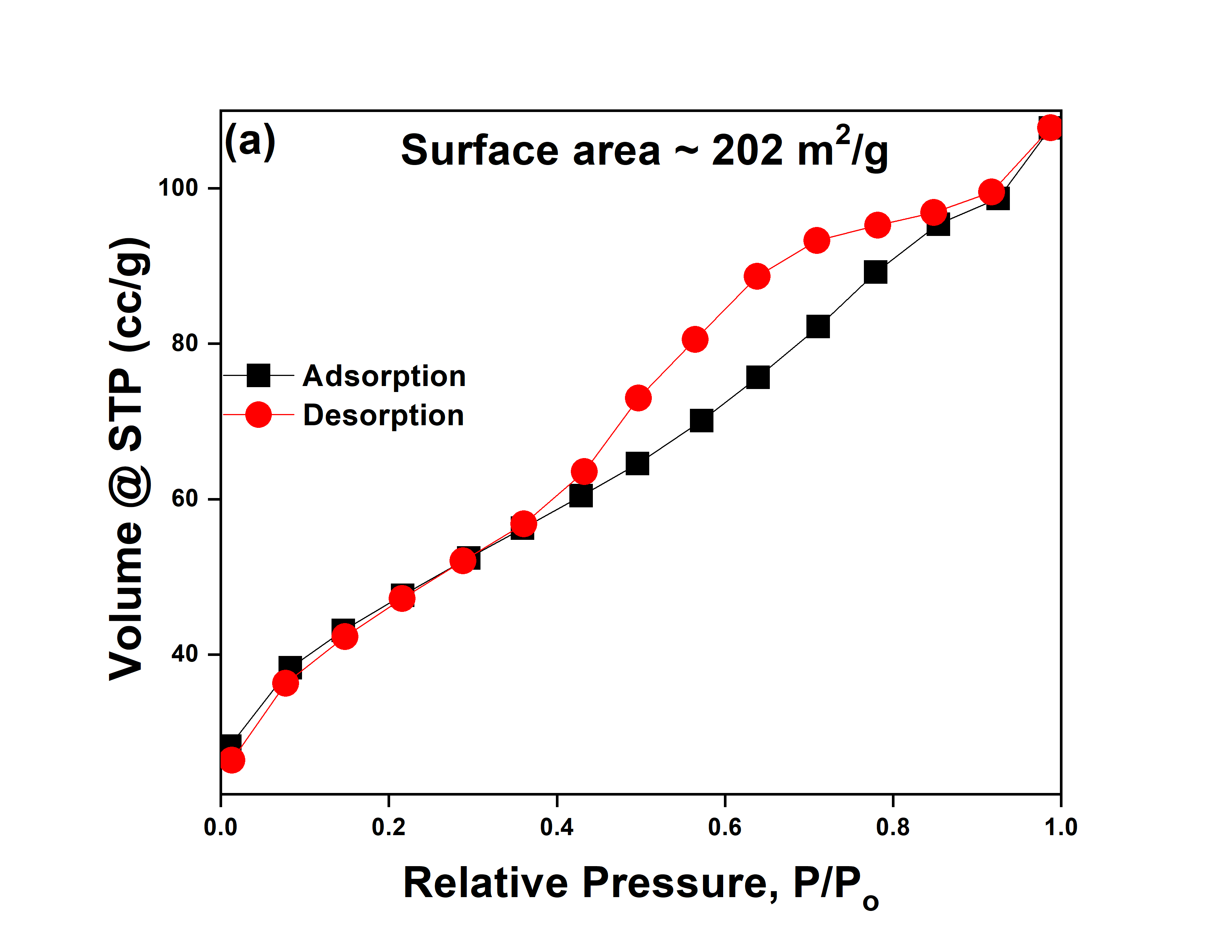

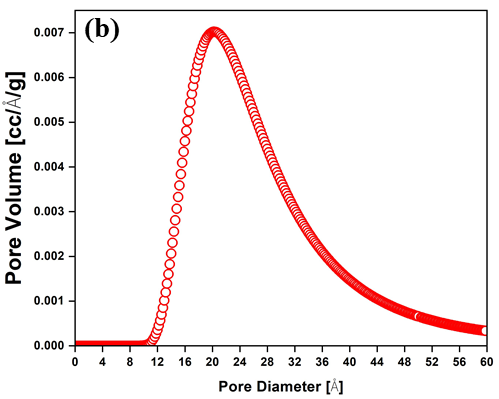


**Fig. S5** (a) N_2_ adsorption/desorption isotherms and the pore size distribution of the mesoporous Ti_0.7_W_0.3_O_2_ catalyst support

The N_2_ adsorption/desorption isotherms were performed to examine the surface area of the Ti_0.7_W_0.3_O_2_ supports. **Fig. S5a** indicated the Ti_0.7_W_0.3_O_2_ support possessed the hysteresis loops of the type IV isotherm, suggesting the as-synthesized Ti_0.7_W_0.3_O_2_ NPs is the mesoporous materials with a mean pore size of ~2.02 nm (**Fig. S5b**). The surface area of the mesoporous Ti_0.7_W_0.3_O_2_ supports was found to be around 202 m^2^/g, which is comparable to the surface area of the carbon black (~230 m^2^/g) and higher than those of other non-carbon support in the previous studies (**Table S1**). Besides the surface area, the electronic conductivity is also the key requirements of the support materials for Pt-based electrocatalysts. The four-point probe technique was used to record the electrical conductivity of Ti_0.7_W_0.3_O_2_ and Vulcan XC-72 supports at the same measurement condition. The electrical conductivity of Vulcan XC-72 support in this work was ~2.0 S/cm, corresponding to the conductivity of Vulcan XC-72 support in the previous study^6^. The electrical conductivity of Ti_0.7_W_0.3_O_2_ support was ~2.2x10^-2^ S/cm, which was much higher than those of other non-carbon supports in the previous studies (**Table 1**) and met the requirement for support materials in fuel cells^7^.

**Table S1.** Properties of the mesoporous Ti_0.7_W_0.3_O_2_ catalyst support and other non-carbon support catalyst support

| **Supports** | **Structure** | **Particle size (nm)** | **Surface area (m^2^/g)** | **Electrical conductivity (S/cm)** | **Ref** |
| --- | --- | --- | --- | --- | --- |
| **Ti_0.7_W_0.3_O_2_** | **Anatase** | **~9.00** | **~202** | **0.022** | **This work** |
| Ti_0.7_Ir_0.3_O_2_ | Rutile | 70-80 nm (length)  25-30 nm (diameter) | 71.123 | 0.028 | ^8^ |
| Ti_0.7_Mo_0.3_O_2_ | Anatase | 8 – 10 | 230 | 2.8x10^-4^ | ^7^ |
| Ti_0.7_W_0.3_O_2_ | Rutile | Agglomeration | - | 0.020 | ^9^ |
| Ti_0.7_Nb_0.3_O_2_ | Anatase + Rutile | - | 46 | 0.0014 | ^10^ |
| Ti_0.7_Ta_0.3_O_2_ | Rutile | 67 | 26 | 0.209 | ^11^ |
| Ta_0.08_Nb_0.2_Ti_0.72_O_2_ | Rutile | - | 8.6 | 8.73x10^-4^ | ^12^ |

**Electrochemical properties of the mesoporous Ti_0.7_W_0.3_O_2_ support**


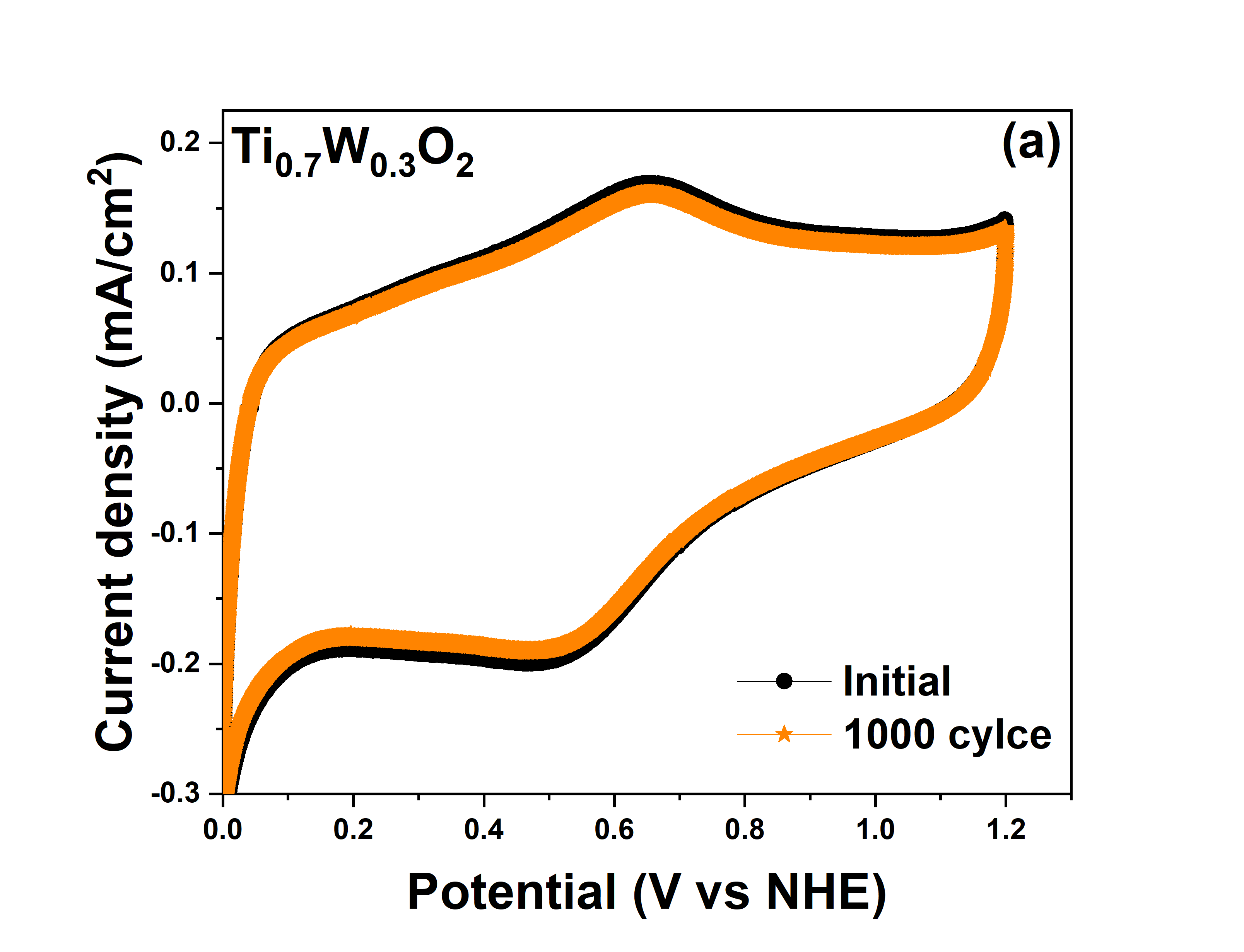

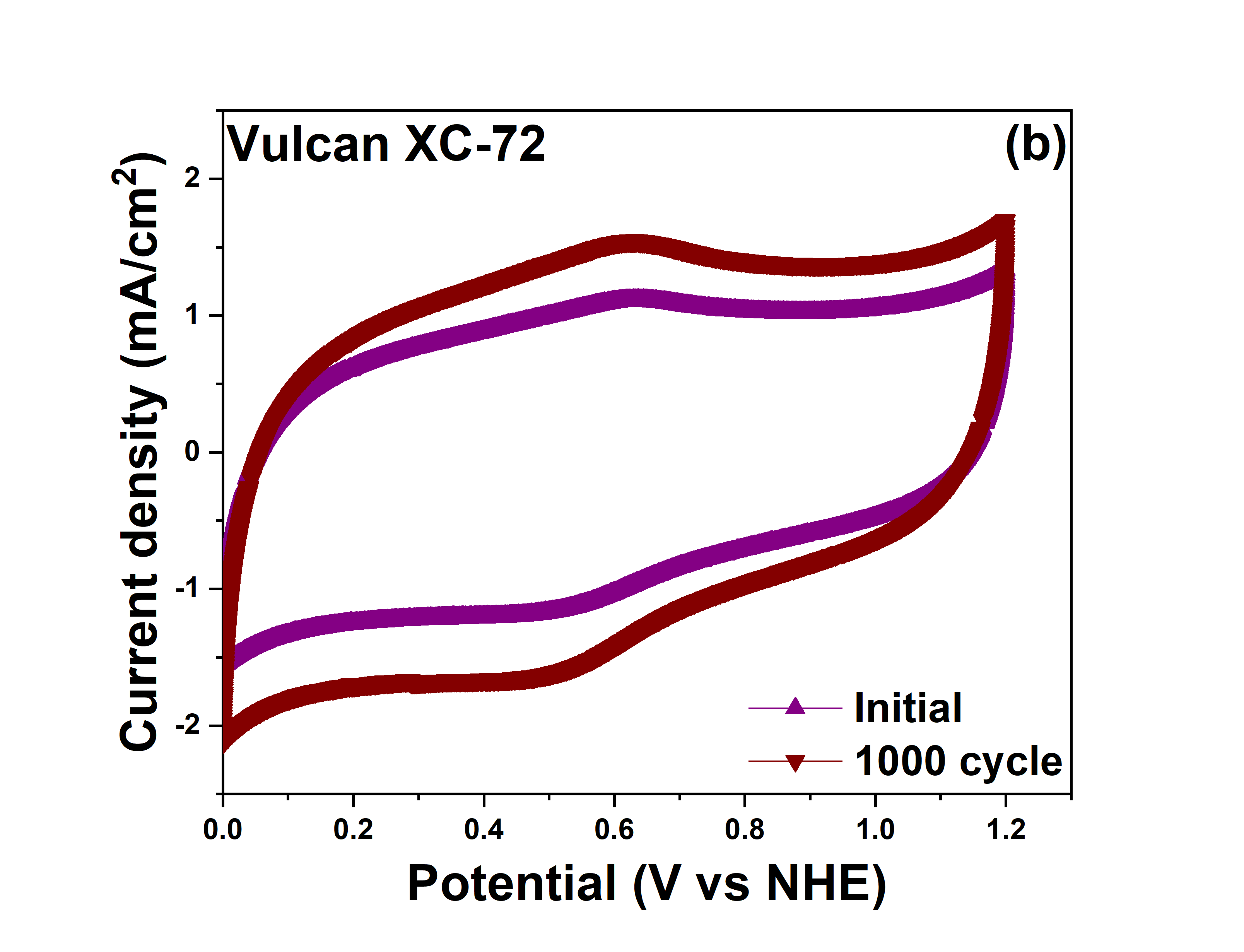


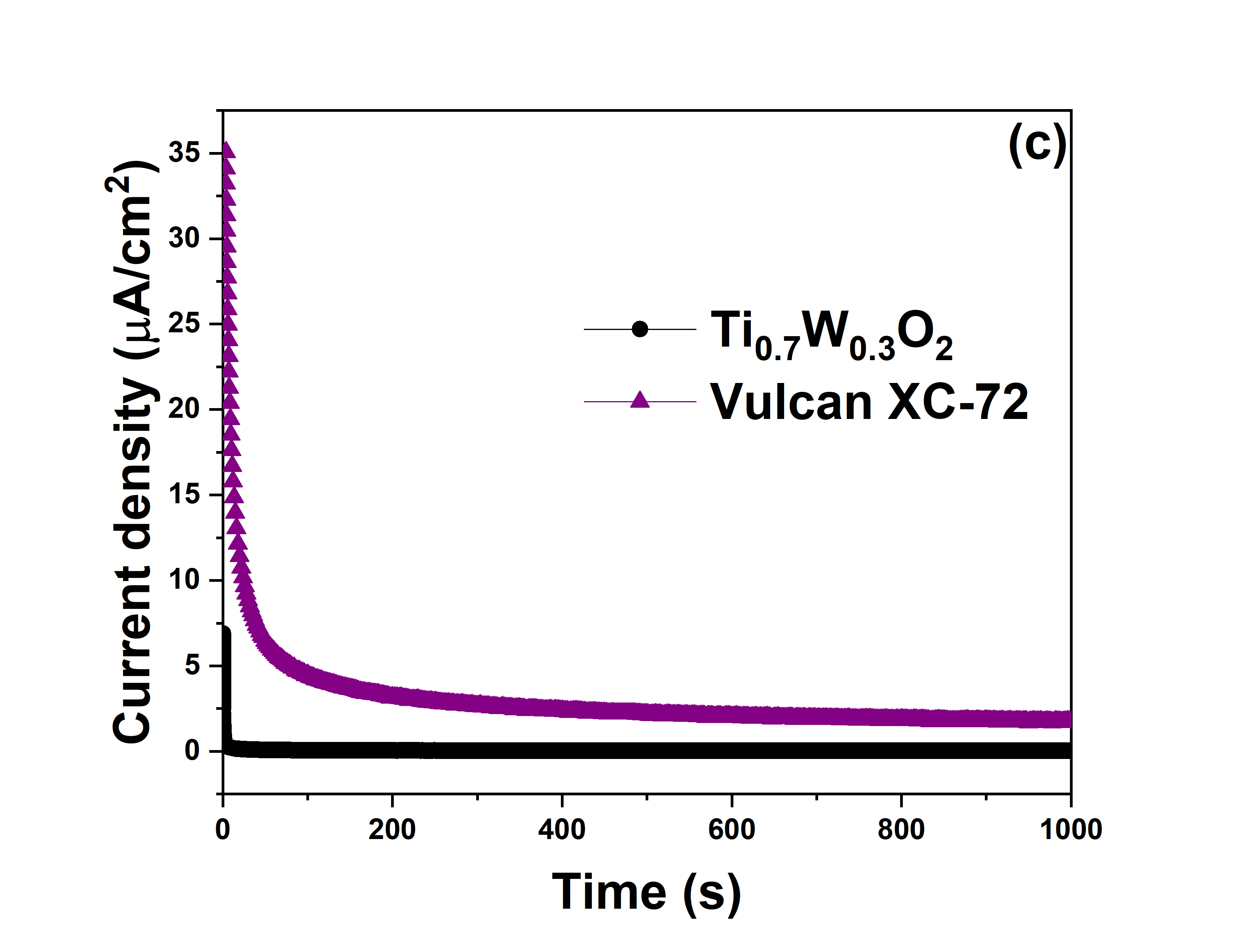


**Fig. S6** Cyclic voltammograms of (a) Ti_0.7_W_0.3_O_2_ catalyst support; (b) Vulcan XC-72 support in N_2_-purged 0.5 M H_2_SO_4_ solution with a scan rate of 50 mV/s; (c) chronoamperometric curves in N_2_-purged 0.5 M H_2_SO_4_ solution at an immobilized potential of 1.6 V for 1000 s

Until now, the poor electrochemical durability of the carbon-based support is still a restriction for large-scale commercialization of fuel cells. The electrochemical stability of the mesoporous Ti_0.7_W_0.3_O_2_ support was investigated and compared to the Vulcan XC-72 by means of the accelerated durability test (ADT) in N_2_-purged 0.5 M H_2_SO_4_ solution at a scan rate of 50 mV/s. **Fig. S6** indicated that the mesoporous Ti_0.7_W_0.3_O_2_ support exhibited the superior electrochemical stability versus that of the Vulcan XC-72 support. For instances, after 1000 cycling test, the CV curves of the mesoporous Ti_0.7_W_0.3_O_2_ support are not almost changed, meanwhile, the CV curves of the Vulcan XC-72 support show the significant change, which is attributable to the corrosion of the Vulcan XC-72 support in acidic media^13-16^ (see **Fig. S6 (a,b)**). Furthermore, the chronoamperometry measurement in N_2_-purged 0.5 M H_2_SO_4_ solution for 1000 s at the immobilized potential of 1.6 V, which was chosen because of it is close to the anode potential under the condition of fuel starvation ^17^. **Fig. S6c** indicated that the corrosion current of Ti_0.7_W_0.3_O_2_ catalyst support was found to be around 0.15 µA/cm^2^, which 12-folds lower than that of the Vulcan XC-72 support (~1.76 µA/cm^2^). The high electrochemical stability of the Ti_0.7_W_0.3_O_2_ support could be ascribed to the inherent structural and chemical durability and the corrosion resistance of the TiO_2_-based oxide in an acidic and oxidative environment.^7^

**Characterization of the Pt NWs/Ti_0.7_W_0.3_O_2_ electrocatalyst**


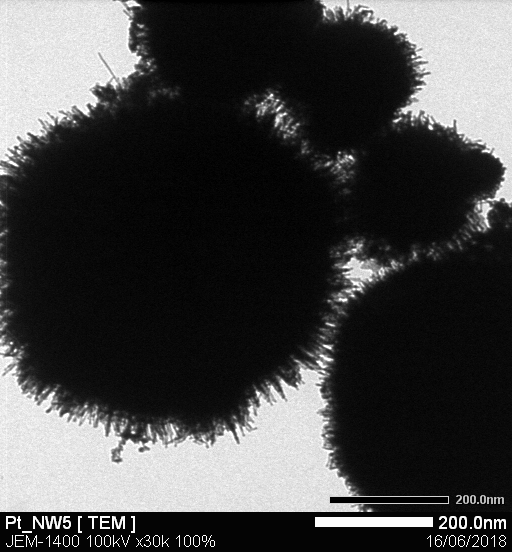
**
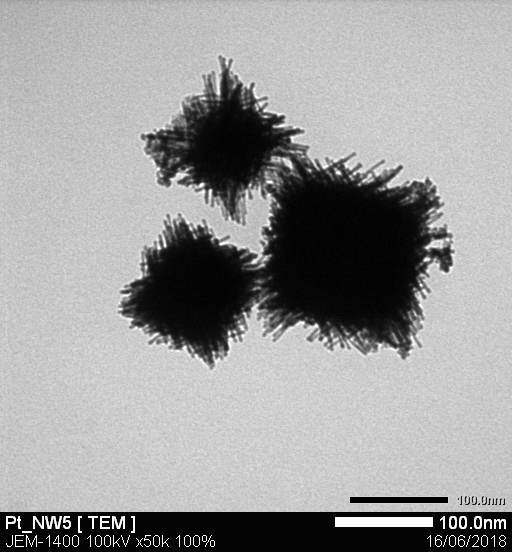
**

**Fig. S7** TEM images of the Pt NWs/Ti_0.7_W_0.3_O_2_ electrocatalyst


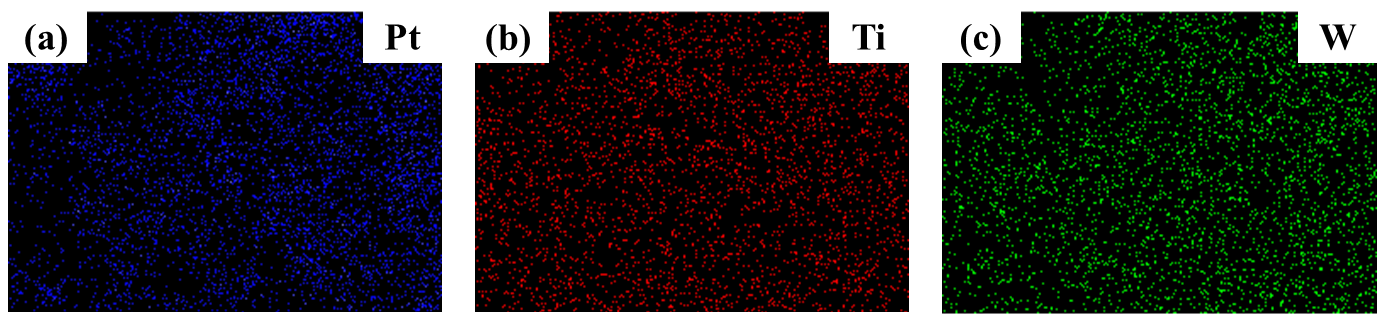


**Fig. S8** Elemental mapping of the Pt NWs/Ti_0.7_W_0.3_O_2_ electrocatalyst

**References**

1. Pham, H. Q.; Huynh, T. T.; Van Nguyen, A.; Van Thuan, T.; Bach, L. G.; Thanh Ho, V. T., Advanced Ti_0.7_W_0.3_O_2_ Nanoparticles Prepared via Solvothermal Process Using Titanium Tetrachloride and Tungsten Hexachloride as Precursors. *J Nanosci Nanotechnol* **2018,** *18* (10), 7177-7182.

2. Huynh, T. T.; Pham, H. Q.; Van Nguyen, A.; Ngoc Mai, A. T.; Nguyen, S. T.; Bach, L. G.; Vo, D.-V. N.; Thanh Ho, V. T., High conductivity and surface area of Ti_0.7_W_0.3_O_2_ mesoporous nanostructures support for Pt toward enhanced methanol oxidation in DMFCs. *International Journal of Hydrogen Energy* **2018**.

3. Belver, C.; Han, C.; Rodriguez, J. J.; Dionysiou, D. D., Innovative W-doped titanium dioxide anchored on clay for photocatalytic removal of atrazine. *Catalysis Today* **2017,** *280*, 21-28.

4. Liu, S.; Guo, E.; Yin, L., Tailored visible-light driven anatase TiO2 photocatalysts based on controllable metal ion doping and ordered mesoporous structure. *Journal of Materials Chemistry* **2012,** *22* (11).

5. Gao, B.; Ma, Y.; Cao, Y.; Yang, W.; Yao, J., Great Enhancement of Photocatalytic Activity of Nitrogen-Doped Titania by Coupling with Tungsten Oxide. *The Journal of Physical Chemistry B* **2006,** *110* (29), 14391-14397.

6. Chinmayee V. Subban, Q. Z., Anthony Hu, Thomas E. Moylan, Frederick T. Wagner, and Francis J. DiSalvo Sol-Gel Synthesis, Electrochemical Characterization, and Stability Testing of Ti_0.7_W_0.3_O_2_ Nanoparticles for Catalyst Support Applications in Proton-Exchange Membrane Fuel Cells. *J. Am. Chem. Soc.* **2010,** *132*, 17531–17536

7. Ho, V. T.; Pan, C. J.; Rick, J.; Su, W. N.; Hwang, B. J., Nanostructured Ti_0.7_Mo_0.3_O_2_ support enhances electron transfer to Pt: high-performance catalyst for oxygen reduction reaction. *J Am Chem Soc* **2011,** *133* (30), 11716-24.

8. Nguyen, A. V.; Huynh, T. T.; Pham, H. Q.; Thi Phan, V. T.; Nguyen, S. T.; Ho, V. T. T., Novel nanorod Ti_0.7_Ir_0.3_O_2_ prepared by facile hydrothermal process: A promising non-carbon support for Pt in PEMFCs. *International Journal of Hydrogen Energy* **2018**.

9. Zheng, L.; Xiong, L.; Liu, Q.; Xu, J.; Kang, X.; Wang, Y.; Yang, S.; Xia, J.; Deng, Z., Facile preparation of rutile Ti_0.7_W_0.3_O_2_ with high conductivity and its effect on enhanced electrocatalytic activity of Pt as catalyst support. *Electrochimica Acta* **2014,** *150*, 197-204.

10. Nguyen, S. T.; Yang, Y.; Wang, X., Ethanol electro-oxidation activity of Nb-doped TiO_2_ supported PdAg catalysts in alkaline media. *Applied Catalysis B: Environmental* **2012,** *113-114*, 261-270.

11. Kumar, A.; Ramani, V., Ta_0.3_Ti_0.7_O_2_ Electrocatalyst Supports Exhibit Exceptional Electrochemical Stability. *Journal of The Electrochemical Society* **2013,** *160* (11), F1207-F1215.

12. Wang, Y.-J.; Wilkinson, D. P.; Neburchilov, V.; Song, C.; Guest, A.; Zhang, J., Ta and Nb co-doped TiO_2_ and its carbon-hybrid materials for supporting Pt–Pd alloy electrocatalysts for PEM fuel cell oxygen reduction reaction. *Journal of Materials Chemistry A* **2014,** *2* (32), 12681-12685.

13. Lv, H.; Mu, S., Nano-ceramic support materials for low temperature fuel cell catalysts. *Nanoscale* **2014,** *6* (10), 5063-5074.

14. Li, Y.; Zhang, X.; Wang, S.; Sun, G., Durable Platinum-Based Electrocatalyst Supported by Multiwall Carbon Nanotubes Modified with CeO_2_. *ChemElectroChem* **2018,** *5* (17), 2442-2448.

15. Wang, H.; Ma, Q., WO_3_-N-doped Carbon Supported PtSn Nanoparticles for Improved Ethanol Oxidation. *Journal of The Electrochemical Society* **2014,** *161* (12), F1202-F1207.

16. Avasarala, B.; Moore, R.; Haldar, P., Surface oxidation of carbon supports due to potential cycling under PEM fuel cell conditions. *Electrochimica Acta* **2010,** *55* (16), 4765-4771.

17. Dou, M.; Hou, M.; Zhang, H.; Li, G.; Lu, W.; Wei, Z.; Shao, Z.; Yi, B., A highly stable anode, carbon-free, catalyst support based on tungsten trioxide nanoclusters for proton-exchange membrane fuel cells. *ChemSusChem* **2012,** *5* (5), 945-51.
